# ErbB2 Receptor Over-Expression Improves Post-Traumatic Peripheral Nerve Regeneration in Adult Mice

**DOI:** 10.1371/journal.pone.0056282

**Published:** 2013-02-21

**Authors:** Giulia Ronchi, Giovanna Gambarotta, Federica Di Scipio, Paolina Salamone, Andrea E. Sprio, Federica Cavallo, Isabelle Perroteau, Giovanni N. Berta, Stefano Geuna

**Affiliations:** 1 Department of Clinical and Biological Sciences, University of Turin, Orbassano (TO), Italy; 2 Neuroscience Institute of the “Cavalieri Ottolenghi” Foundation (NICO), University of Turin, Orbassano (TO), Italy; 3 Molecular Biotechnology Center, University of Turin, Turin, Italy; King’s College London, United Kingdom

## Abstract

In a transgenic mice (BALB-neuT) over-expressing ErbB2 receptor, we investigated the adult mouse median nerve in physiological and pathological conditions. Results showed that, in physiological conditions, the grip function controlled by the median nerve in BALB-neuT mice was similar to wild-type (BALB/c). Stereological assessment of ErbB2-overexpressing intact nerves revealed no difference in number and size of myelinated fibers compared to wild-type mice. By contrast, after a nerve crush injury, the motor recovery was significantly faster in BALB-neuT compared to BALB/c mice. Moreover, stereological assessment revealed a significant higher number of regenerated myelinated fibers with a thinner axon and fiber diameter and myelin thickness in BALB-neuT mice. At day-2 post-injury, the level of the mRNAs coding for all the ErbB receptors and for the transmembrane (type III) Neuregulin 1 (NRG1) isoforms significantly decreased in both BALB/c and BALB-neuT mice, as shown by quantitative real time PCR. On the other hand, the level of the mRNAs coding for soluble NRG1 isoforms (type I/II, alpha and beta) increased at the same post-traumatic time point though, intriguingly, this response was significantly higher in BALB-neuT mice with respect to BALB/c mice. Altogether, these results suggest that constitutive ErbB2 receptor over-expression does not influence the physiological development of peripheral nerves, while it improves nerve regeneration following traumatic injury, possibly through the up-regulation of soluble NRG1 isoforms.

## Introduction

Peripheral nerve regeneration after a traumatic injury is regulated by the combined action of many factors [1]. Investigating the mechanisms underlying posttraumatic nerve regeneration is of great relevance because it can lead to effective new regenerative strategies to improve patient outcome after nerve reconstruction [2]. Over the past years, studies on nerve regeneration have increasingly employed the use of transgenic mice, leading to a better comprehension of nerve regenerative processes after trauma [3], [4].

Schwann cells are the key element for the promotion of axonal regeneration [1], [5], [6] and thus identifying the signals that control Schwann cell response to nerve injury is of great biological and clinical interest. One interesting candidate for the regulation of Schwann cell proliferation is the tyrosine kinase receptor ErbB2 (also called HER-2 or Neu), which belongs to the epidermal growth factor (EGF) receptor family [7], [8], [9], [10]. This receptor is involved in the signal transduction pathways leading to physiologic processes, such as embryogenesis, cell proliferation, and apoptosis [11] and in regenerative processes concerning nerve [12], heart [13], pancreas [14]. However, its deregulation can drive cancer development and progression in some histotypes; from this point of view ErbB2 is an oncogene having a role as a negative prognostic marker [15], [16], [17] and as a target for antineoplastic therapy [18], [19], [20], [21]. No ErbB2 ligands have been identified yet. However, ligand binding to other ErbB family members induces heterodimerization and activation of ErbB2. Genetic evidence shows that ErbB2 participates in the transduction of signals downstream a family of ligands of the EGF family known as neuregulins [22].

The ligand of the ErbB2/ErbB3 heterodimer is the neuregulin-1 (NRG1), which has a key role in the development of the peripheral nervous system. In mammals, there are several isoforms of NRG1, which can be soluble (type I/II) or transmembrane (type III), with different spatiotemporal patterns of expression [23], [24], [25], [26]. It has been shown that juxtacrine and paracrine signaling mediated by transmembrane and soluble NRG1 isoforms play different roles during Schwann cell myelination and repair [27], [28], [29]; axons lacking NRG1 show a slower rate of regeneration with impaired remyelination [30].

In the present study, we investigated in the median nerve the effects of ErbB2 over-expression; median nerves from mice of the same offspring carrying (BALB-neuT mice) or not (BALB/c mice) the rat neu/ErbB2 gene under the control of the mouse mammary tumor virus (MMTV) promoter, were analyzed in both physiological conditions and after crush injury.

## Materials and Methods

### Mice and Surgery

6-week old male BALB/c and BALB-neuT mice from the same offspring were kindly provided by Prof. G. Forni (Molecular Biotechnology Center, University of Turin). All these mice were born from the mating of BALB-neuT male mice carrying the rat ErbB2 transgene under the control of the mouse mammary tumor virus (MMTV) promoter, in heterozygosis with wild-type BALB/c females [31]. Following genotyping for rat ErbB2 mice, the offspring was divided in wild type (BALB/c) mice and transgenic heterozygous (BALB-neuT) mice, over-expressing ErbB2. Only male mice were studied since BALB-neuT female mice develop fast growing mammary carcinomas [31], [32]. A total of 32 mice were used for this study: 4 mice were used for the western blot analysis, 10 mice were used for the grasping test, morphological and stereological analysis (5 BALB/c and 5 BALB-neuT) and 18 mice were used for the quantitative real time PCR analysis (9 BALB/c and 9 BALB-neuT).

All mouse experiments were performed with the approval of the local Institution’s Animal Care and Ethics Committee of the University of Turin and in accordance with the European Communities Council Directive of 24 November 1986 (86/609/EEC). Mice were housed in a temperature and humidity controlled room with 12–12 h light/dark cycles and fed with standard chow and water ad libitum. Measures were taken to minimize pain and discomfort taking into account human endpoints for animal suffering and distress.

For the surgery, mice were deeply anaesthetized via intraperitoneal injection with ketamine (9 mg/100 g-body weight), xylazine (1.25 mg/100 g-body weight) and atropine (0.025 mg/100 g-body weight). The median nerve of the left forelimb was approached from the axillary region to the elbow. Under operative microscope, the median nerve was carefully exposed and the crush lesion was performed by compressing the nerve for 30 seconds with a non-serrated clamp (manufactured by the Institute of Industrial Electronic and Material Sciences, University of Technology, Vienna, Austria) [33], [34]. The force applied to the nerve was 61.3 N giving a final pressure of 20.43 Mpa [35]. In order to prevent interferences with the grasping test, the contra-lateral median nerve (right arm) was transected at the middle third of the brachium and its proximal stump was sutured to the pectoralis major muscle to avoid spontaneous reinnervation. The contro-lateral uninjured median nerves were collected and used as control. We therefore obtained four experimental groups: 1) uninjured BALB/c nerves; 2) uninjured BALB-neuT nerves; 3) injured BALB/c nerves; 4) injured BALB-neuT nerves.

For quantitative real time PCR analysis three independent experiments were performed. For each experiment, six mice were used: three BALB/c and three BALB-neuT mice. To obtain an adequate amount of RNA, all animals underwent a unilateral crush lesion on the median, ulnar and radial nerves following the same protocol described above.

### Postoperative Assessment of Functional Recovery

Grasping test was carried out weekly starting from the day before the surgery until day-28. Grasping test was performed using the BS-GRIP Grip Meter (2 Biological Instruments, Varese, Italy), which is represented by a precision dynamometer connected to a grid. Briefly, the mice were elevated by the tail and allowed to grasp the grid [36]. The dynamometer records the maximum weight that the mouse manages to hold up before losing the grip. The maximal strength monitored by the dynamometer is an indicator of muscle function. For each time point, each mouse was tested three times and the average value was recorded.

### Immunocytochemistry

Median nerves from uninjured BALB/c and BALB-neuT mice were fixed in 4% paraformaldehyde for 2 h, washed in a solution of 0.01 M PBS (pH 7.2) for 30 min, dehydrated, and embedded in paraffin. Sections were cut 8–10 µm thick, permeabilized, blocked [0.1% triton X-100, 10% normal goat serum (NGS)/0.02% NaN3, 1 h] and processed for an immunohistochemical protocol. Mouse monoclonal primary antibody against ErbB2 (dilution 1∶40, Oncogene), rabbit polyclonal primary antibody against GFAP (dilution 1∶600, Sigma) and mouse monoclonal primary antibody against tubulin βIII (dilution 1∶1000, Sigma) were applied overnight at room temperature for single or double staining. Subsequently, the sections were incubated for 1 hour at room temperature with goat α-rabbit IgG Cy3 (dilution 1∶200 Jackson ImmunoResearch) and/or goat α-mouse Alexa488 (dilution 1∶200, Molecular Probes). The samples were finally mounted with a Dako fluorescent mounting medium and analyzed by a LSM 510 confocal laser microscopy system (Zeiss, Jena, Germany).

### Resin Embedding, High-resolution Light Microscopy and Electron Microscopy

Under deep anesthesia, mice were sacrificed at day-28 post-injury and 5-mm long segment of the median nerve distal to the site of the crush lesion from both BALB/c and BALB-neuT mice was removed. A 4/0 stitch was used to mark the proximal stump of the nerve segment. Nerve samples were fixed and embedded as previously described [37].

Transversal 2.5 µm cross sections were obtained ∼2 to 2.5 mm distal to the lesion site of the withdrawn median nerve sample using an Ultracut UCT ultramicrotome (Leica Microsystems, Wetzlar, Germany) and stained by toluidine blue for high-resolution light microscopy examination and design-based quantitative morphology. Photomicrographs were taken using a DM4000B microscope equipped with a DFC320 digital camera (Leica Microsystems, Wetzlar, Germany) and slightly adjusted for brightness and contrast to obtain uniform plates.

Electron microscopy was performed on the same specimens used for high-resolution light microscopy. Ultra-thin sections (70-nm thick) were cut immediately after the series of semithin section with the same ultramicrotome and double stained with saturated aqueous solution of uranyl acetate and lead citrate. Ultra-thin sections were analyzed using a JEM-1010 transmission electron microscope (JEOL, Tokyo, Japan) equipped with a Mega-View-III digital camera and a Soft-Imaging-System (SIS, Munster, Germany).

### Design-based Quantitative Morphology of Nerve Fiber Regeneration

Design-based quantitative morphology was carried out on one randomly selected toluidine blue stained semi-thin section.

Stereological analysis of myelinated nerve fibers was carried out using a protocol previously described [38], [39], [40]. On the randomly selected section, the total cross-sectional area of the nerve was measured and then an adequate number of fields (6–8) were selected using a systematic random sampling protocol [38], [41].

In each sampling field, the “edge effect” was avoided by employing a two-dimensional dissector procedure which is based on sampling the “tops” of fibers [42], [43].

Mean fiber density was then calculated by dividing the total number of nerve fibers within the sampling field (N) by its area (N/mm^2^). The total number of fibers was estimated by multiplying the mean fiber density by the total cross-sectional area of the whole nerve cross section. In addition, in each fiber, both fiber and axon area were measured and the fiber (D), axon (d) diameter, myelin thickness 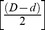
and *g-*ratio 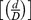 were calculated.

Hence, we analyze the *g-*ratio/axon diameter correlation of individual fibers by means of scatterplots, evaluating the differences in linear regression. Furthermore, we deepen that analysis by means of predictive inference: considering uninjured group *g-*ratio/axon diameter scatterplot as reference, we highlight a graphical area bounded by 95% prediction interval of regression line and ±2 σ of axon diameter. Statistically, about 90% of functional myelinated fibers will fall inside this predictive area.

At the electron microscopic level, the number of Schwann cells was quantified by counting the nuclear profiles numbers in a defined area at 8000×magnification (12,2×16,2 µm^2^). The “edge effect” was avoided according to the stereological method previously described [42], [43].

### Total Protein Extraction and Western Blot Analysis

Total proteins were obtained from uninjured and crushed (2 days post-injury) BALB/c and BALB-neuT mice sacrificed under deep anesthesia. A 6 mm segment of median, ulnar and radial nerves just proximal and distal to the crushed site were harvested for each animal.

Total proteins were extracted in boiling Laemmli buffer (2.5% SDS, 0.125 M Tris-HCl, pH 6.8), followed by 3 min denaturation at 100°C. Protein concentration was determined by the BCA method, and equal amounts of proteins (50 µg) were denaturated 3 min at 100°C in 240 mM beta-mercaptoethanol, 18% glycerol, loaded onto each lane, separated by SDS-PAGE, transferred to a HybondTM C Extra membrane (Amersham Biosciences, General Electric Healthcare Europe) and analyzed as previously described [44]. The western blot was decorated with a polyclonal primary antibody anti ErbB2 (Neu (C-18) # sc-284, 1∶500 w.d., Santa Cruz Biotechnology, Inc., Santa Cruz, CA, USA) and the horseradish peroxidase-linked donkey anti-rabbit secondary antibody (#NA934, 1∶10.000 w.d., Amersham Biosciences).

### RNA Extraction and Quantitative Real-time PCR

To obtain suitable amount of material, total RNA was extracted from pools of median, ulnar and radial nerves of three mice for each of three independent experiments. Under deep anesthesia, mice were sacrificed at day-2 post-injury. A 6 mm segment of the nerves just proximal and distal to the crushed site were harvested for each animal. Total RNA was isolated using the RNeasy Mini Kit (Qiagen S.r.l., Milano, Italy) according to the manufacturer’s protocol.

0.5 µg total RNA were subjected to a reverse transcriptase (RT) reaction in 25 µl reaction volume containing: 1× RT-Buffer (Fermentas, Burlington, Canada); 0.1 µg/µl bovine serum albumin (BSA, Sigma); 0.05% Triton X-100; 1 mM dNTPs; 7.5 µM random exanucleotide primers (Fermentas); 1 U/µl RIBOlock (Fermentas) and 200 U RevertAid™ M-MuLV reverse transcriptase (Fermentas). The reaction was performed for 10 min at 25°C, 90 min at 42°C, 10 min at 90°C.

Quantitative real-time PCR for ErbB1, ErbB2, ErbB3, ErbB4, NRG1-alpha, NRG1-beta, NRG1-I/II and NRG1-III was performed using an ABI Prism 7300 (Applied Biosystems, Life Technologies Europe BV, Monza, Italy) detection system using SYBR Green chemistry. cDNA was diluted 10 times before analysis and 5 µl were analyzed in a total volume of 25 µl containing 1× PowerGREEN Master Mix (Applied Biosystems), and 300 nM of each primer. The reactions were carried out in 40 cycles (primer annealing temperature: 60°C). For each cDNA sample, three technical replicates were averaged and dissociation curves were routinely performed to check for the presence of a single peak corresponding to the required amplicon. Normalized reporter fluorescence (Rn) for each cycle was obtained by normalizing SYBRGreen to ROX signal.

The data from the real-time PCR experiments were analyzed using the ΔΔCt method for the relative quantification. The threshold cycle number (Ct) values of both the calibrator and the samples of interest were normalized to the geometric average of six endogenous housekeeping genes: Ubiquitin C (UbC), TATA box binding protein (TBP), 18S ribosomal RNA (18S rRNA), glyceraldehyde-3-phosphate dehydrogenase (GAPDH), hypoxanthine phosphoribosyltransferase 1 (HPRT1) and the signaling molecule mitogen-activated protein kinase 6 (MAPK6). As calibrator we used the RNA obtained from a pool of uninjured nerves.

**Figure 1:**
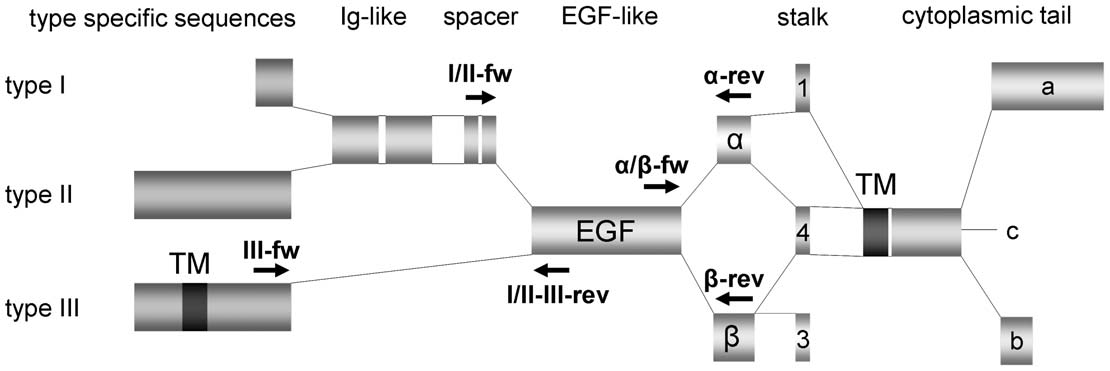
Neuregulin1 primer design.

Primers were designed using Annhyb software (http://www.bioinformatics.org/annhyb/) and synthesized by Invitrogen (Life Technologies Europe BV, Monza, Italy). Primers sequences are reported in Table 1. Neuregulin1 primers design was made as summarized in Figure 1.

**Table 1:** Primers for Real Time PCR (m = Mus musculus; r = Rattus norvegicus).

		Mus musculus	Rattus norvegicus	
primer name	primer sequence (5′-3′)	accession number	accession number	size (bp)
**mErbB1-fw**	GCTGGTGTTGCTGACCGCG	NM_207655.2		86
**mErbB1-rev**	GGGTGAGCCTGTTACTTGTGCC	NM_207655.2		
**rErbB2-fw**	TGACAAGCGCTGTCTGCCG		NM_017003	106
**rErbB2-rev**	CTTGTAGTGGGCGCAGGCTG		NM_017003	
**mErbB2-fw**	GCAAGCACTGTCTGCCATGC	NM_001003817		96
**mErbB2-rev**	GGGCACAAGCCTCACACTGG	NM_001003817		
**mrErbB3-fw**	CGAGATGGGCAACTCTCAGGC	NM_010153	NM_017218.2	129
**mrErbB3-rev**	AGGTTACCCATGACCACCTCACAC	NM_010153	NM_017218.2	
**mrErbB4-fw**	CATGGCCTTCCAACATGACTCTGG	NM_010154.1	AY375307.1	108
**mrErbB4-rev**	GGCAGTGATTTTCTGTGGGTCCC	NM_010154.1	AY375307.1	
**mNRG1-I/II-fw**	CATGTCAGCCTCAACTGAAAGACCC	AY648976.1		116
**mNRG1-III-fw**	CCCTGAGGTGAGAACACCCAAGTC	NM_178591		104
**mNRG1-I/II-III-rev**	TGGTCCCAGTCGTGGATGTAGATG	AY648976.1-NM_178591		
**mNRG1-alfa/beta-fw**	GTGCGGAGAAGGAGAAAACTTTC	AY648976.1-NM_178591	AF194438–AF194439	
**mrNRG1-alfa-rev**	TTGCTCCAGTGAATCCAGGTTG	NT_039457	AF194439	114
**mrNRG1-beta-rev**	AACGATCACCAGTAAACTCATTTGG	AY648976.1-NM_178591	AF194438	117
**mHPRT-fw**	GCCCCAAAATGGTTAAGGTTGCAAG	NM_013556		81
**mHPRT-rev**	ATCCAACAAAGTCTGGCCTGTATCC	NM_013556		
**mr18s-fw**	CGATGGTAGTCGCCGTGCC	NR_003278	M11188	84
**mr18s-rev**	CCGTTTCTCAGGCTCCCTCTCC	NR_003278	M11188	
**mMAPK6-fw**	CAGCTGCTGCCGGGGATC	NM_015806.4		74
**mMAPK6-rev**	CGGTCCATGGGGCTGAAAGTC	NM_015806.4		
**mrGAPDH-fw**	CCACCAACTGCTTAGCCCCC	NM_008084	NM_017008	91
**mrGAPDH-rev**	GCAGTGATGGCATGGACTGTGG	NM_008084	NM_017008	
**mUbc-fw**	CCACCAAGAAGGTCAAACAGG	NM_019639.4		93
**mUbc-rev**	CCCATCACACCCAAGAACAAG	NM_019639.4		
**mTBP-fw**	GATCAAACCCAGAATTGTTCTCC	NM_013684.3		106
**mTBP-rev**	GGGGTAGATGTTTTCAAATGCTTC	NM_013684.3		

### Statistical Analysis

Statistical analysis was performed using PASW statistics 18 (SPSS Inc., Chicago, IL, USA). For the values taken from the different time-point assessments of the grasping test, one-way repeated measures analysis of variance (RM-ANOVA) test followed by post hoc multiple pair-wise comparisons using the Student–Neuman–Keuls (SNK) test was used. For both stereological and grasping test data, the N size used in the statistical calculations was the number of animals (n = 5 for each experimental group). Differences in predictive area fitting are evaluated by Chi-Square test considering the number of fibers falling inside the area versus those falling outside.

For quantitative real time PCR data, statistical analysis was performed using the one-way ANOVA test followed by Bonferroni post-hoc multiple comparison test. The interaction between the effect of injury and ErbB2 over-expression was investigated by two-way ANOVA test.

## Results

### BALB-neuT Mice Express Higher Levels of ErbB2 Protein in the Peripheral Nervous System

**Figure 2:**
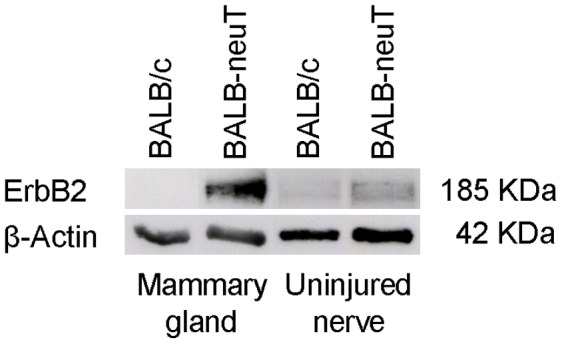
BALB-neuT mice express higher levels of ErbB2 protein in peripheral nerves. A: Western blot analysis showing ErbB2 expression in mammary gland and in uninjured peripheral nerves. β-actin was used as a loading control. Mammary gland from BALB-neuT mice was used as positive control for ErbB2 overexpression. The ErbB2 level is higher in BALB-neuT mice compared to BALB/c mice. B–C.

To study the role of ErbB2 overexpression in physiological conditions and after crush injury of peripheral nerves, we performed a preliminary analysis to evaluate protein expression in BALB-neuT mice carrying the rat neu/ErbB2 gene under the control of the mouse mammary tumor virus (MMTV) promoter. We detected a higher level of ErbB2 protein in the median, ulnar and radial nerves of BALB-neuT compared with BALB/c mice (Fig. 2A).

**Figure 3:**
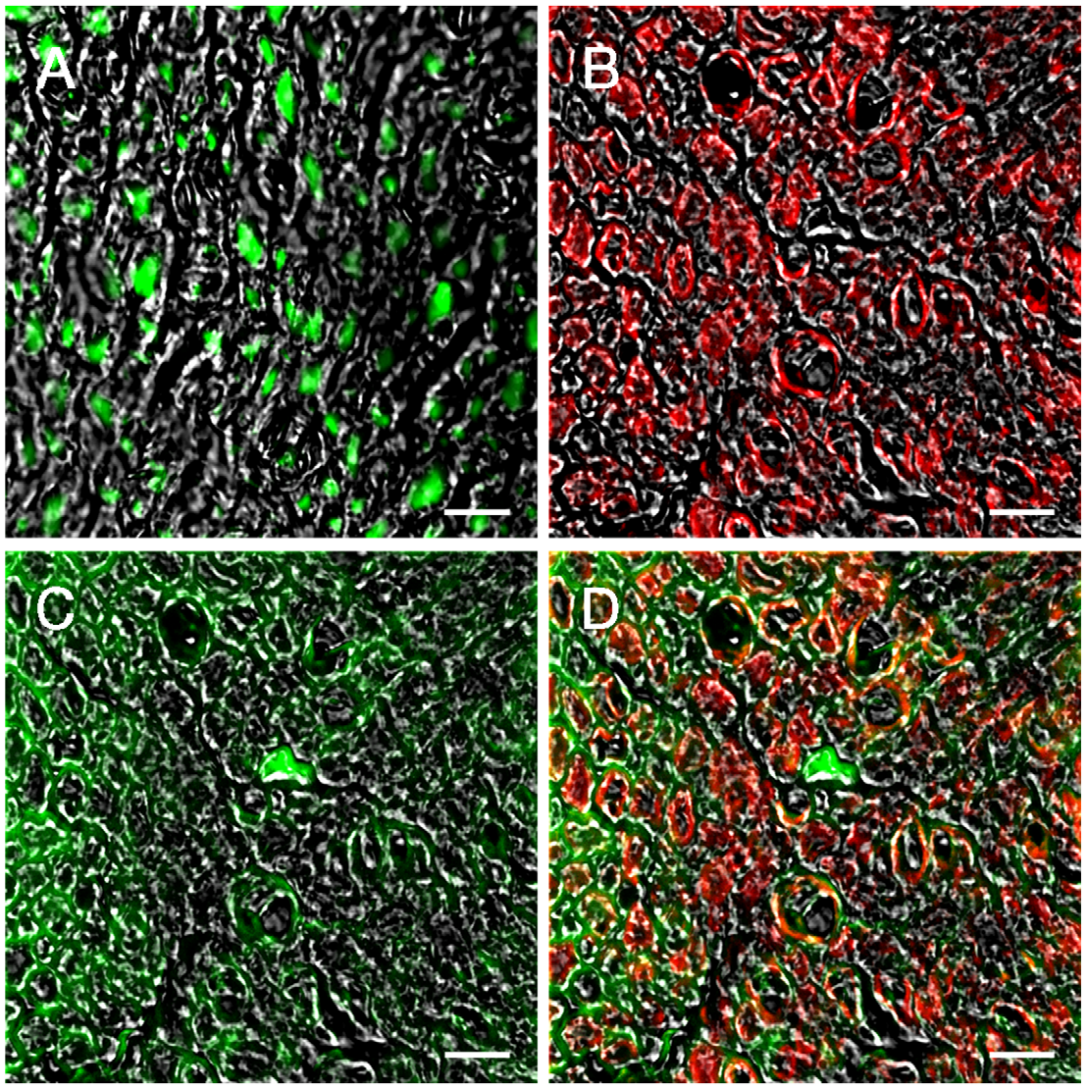
ErbB2 transgene is expressed by Schwann cells. Immunohistochemistry analysis showing the localization of ErbB2 protein in BALB-neuT median merve. A, tubulinβIII staining; B, GFAP staining; C, ErbB2 staining; D: colocalization between GFAP and ErbB2 showing that the ErbB2 protein is expressed by Schwann.Scale bar: 10 µm.

Figure 3 shows immune-labeling for tubulinβIII (A), GFAP (B) and ErbB2 (C). Double labeling with GFAP and ErbB2 protein (D) shows the co-localization between the two markers in Schwann cells (Fig. 3D).

### ErbB2 Over-expression has No Effects on Mice Median Nerve in Physiological Conditions

**Figure 4:**
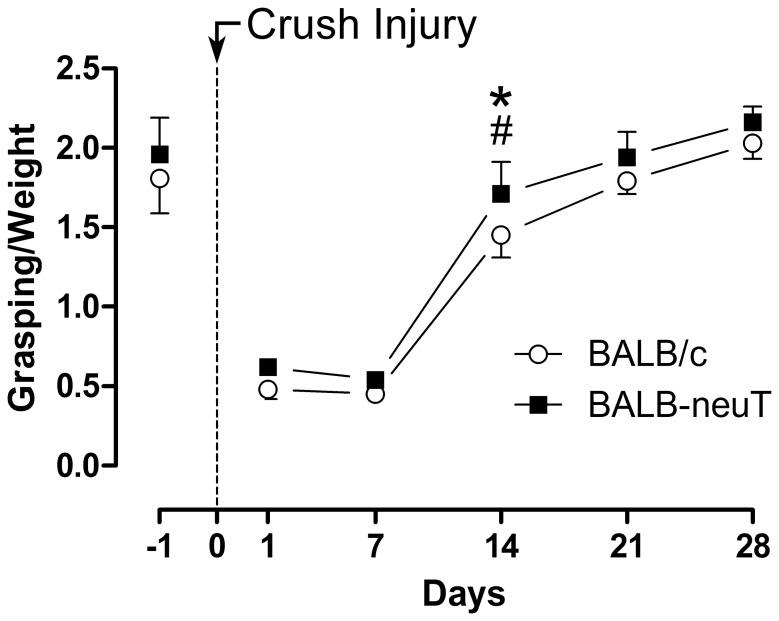
14 days after the injury, grasping strength is greater in BALB-neuT than in BALB/c mice. Graph showing the performance of the mice in the grasping test after normalizing the recorded values with the animal body weights. Data are reported as means ± SEM (*BALB/c vs BALB-neuT; # regenerating vs pre-injury wild-type, p<0.05).

Although BALB-neuT mice weighted significantly (p<0.05) less (23.22 g ±1.38 g at the day of the sacrifice) than the BALB/c (26.92 g ±0.38 g at the day of the sacrifice), they showed no evident behavioral abnormalities. In particular, no signs of motor and sensory impairment were detectable in BALB-neuT compared to BALB/c mice. Motor function was tested by means of the grasping test the day before the injury (Fig. 4, day-1) and no deficits were recorded in transgenic mice.

**Figure 5:**
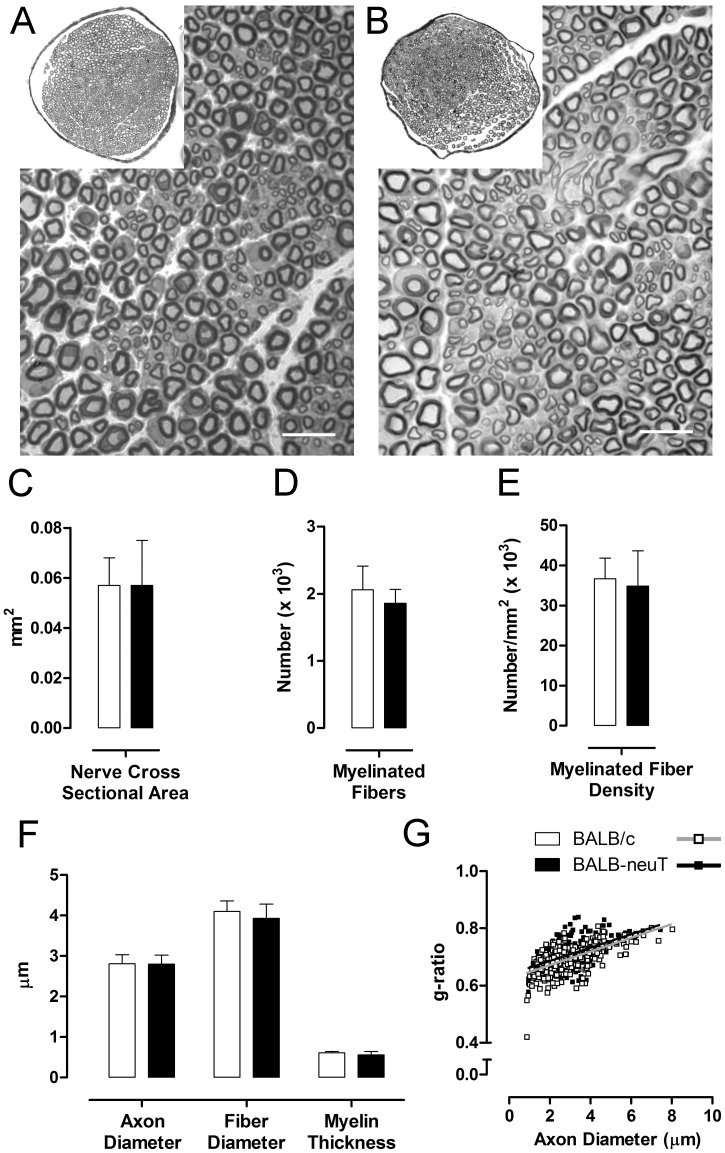
Morphological and stereological analysis of uninjured median nerves do not show detectable differences. A, B: representative light micrographs of transverse sections of median nerves stained with toluidine blue of BALB/c and BALB-neuT mice respectively. In both groups myelinated axons have a normal morphological appearance. Bar = 10 µm. C, D, E, F: histograms showing the results of histomorphometric evaluations. No significant differences are detectable for all analyzed parameters. Values in the graphics are expressed as mean+SEM. G: Scatterplots displaying *g*-ratios of individual fibers in relation to respective axon diameter (obtained from more than 250 myelinated axons per group, 5 mice per genotype) are not different in BALB/c and BALB-neuT mice.

To determine if any peripheral structural parameter correlates with the over-expression of ErbB2, we compared semi-thin sections stained with toluidine blue from the median nerve of BALB/c (Fig. 5A) and BALB-neuT (Fig. 5B) mice. No significant morphological differences were detected between the two groups. Stereological analysis showed that the cross sectional area (Fig. 5C), the number of total myelinated fibers (Fig. 5D) and the myelinated fiber density (Fig. 5E) were not significantly different between BALB/c and BALB-neuT mice. Also the parameters related to the fiber size (axon diameter, fiber diameter and myelin thickness) were not significantly different between BALB/c and BALB-neuT mice (Fig. 5F). This result was further confirmed by *g*-ratio measurement (expressed as scatterplots) of median nerve fibers from BALB/c (0.69±0.01) and BALB-neuT mice (0.71±0.02) (Fig. 5G).

**Figure 6:**
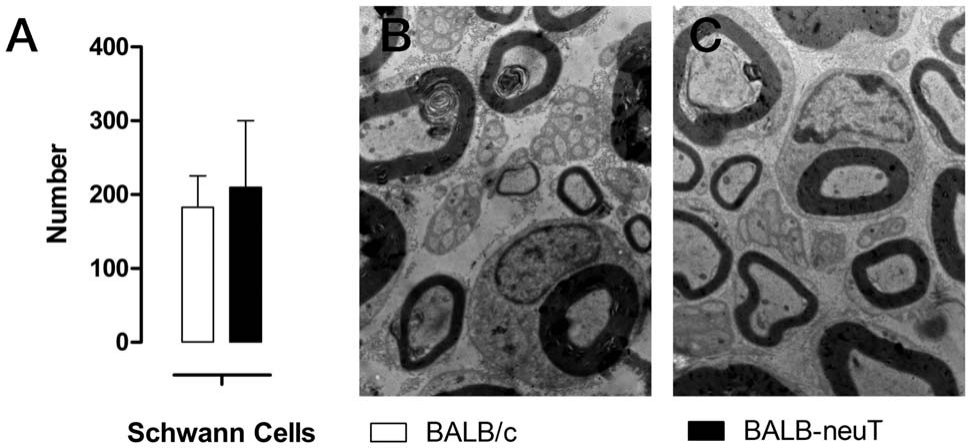
The number of Schwann cells is comparable between BALB/c and BALB-neuT mice. A, histognams showing the number of Schwann cells counted at electron microscopy. B, C representative electron images of transverse sections of uninjured median nerves of BALB/c and BALB-neuT mice respectively. Magnification: 10000×.

We also evaluated the number of Schwann cell nuclei by electron microscopy analysis. No difference was seen in the number and/or morphology of Schwann cells between uninjured BALB/c and uninjured BALB-neuT mice (Fig. 6), as demonstrated by electron microscopy and cell counting.

### ErbB2 Over-expression Improves the Regeneration after Median Nerve Crush Injury

To investigate whether ErbB2 over-expression exerts any effect on axonal regeneration after a median nerve crush injury, we compared nerve injury responses between BALB/c and BALB-neuT mice. Motor functional recovery after the injury was assessed every 7 days until day-28 beginning from the day just after the injury. Results are presented as the ratio between grip strength and animal weight (Fig. 4). From day-1 to day-7 post-injury the fingers of all the mice were in complete extension, but the values did not fall to zero because the weight of the mouse paw was revealed by the balance.

Statistical analysis showed that, at day-14 post-injury, ratio was significantly higher in BALB-neuT mice in comparison to BALB/c supporting thus the view that the progression of functional recovery is faster in relation to ErbB2 over-expression.

Moreover, BALB-neuT mice reached values not significantly different from the pre-injury value already at day-14, while, at this time point, BALB/c group was still statistically different compared to the pre-injury value. At day-21 also BALB/c group reached values not statistically different from the pre-injury condition.

**Figure 7:**
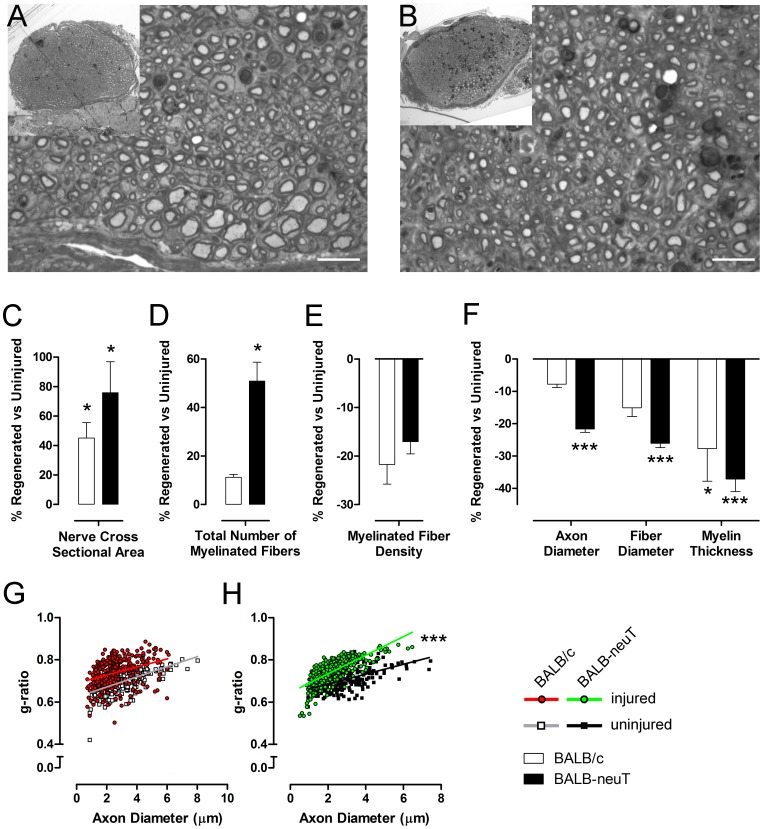
Morphological and stereological analysis of the regenerated median nerves show significant differences. A, B: representative light micrographs of transverse sections of median nerves stained with toluidine blue of BALB/c and BALB-neuT mice, respectively. Bar = 10 µm. C, D, E, F: histograms showing the results of histomorphometric evaluations of nerve regeneration. After the regenerative process, BALB-neuT nerves show more and smaller myelinated fibers compared to uninjured ones. Histograms are represented as percentage variation of regenerated nerves with respect to relative uninjured nerves; values in the graphics are expressed as mean+SEM). G, H: scatterplots displaying *g*-ratios of individual fibers in relation to respective axon diameter (obtained from more than 250 myelinated axons per group, 5 mice per genotype) show that in both BALB/c (G) and BALB-neuT (H) mice *g*-ratio is higher after injury. Statistical significance: *p<0.05, **p<0.01, ***p<0.001.

Semi-thin sections stained with toluidine blue from regenerated median nerve showed that 28 days after the crush injury in both BALB/c and BALB-neuT mice the regeneration was adequate, with the presence of many regrowing fibers organized in microfascicles (Fig. 7A–B). They appeared to be smaller and more numerous compared to uninjured nerves (Fig. 7A–B). Moreover, the amount of connective tissue was greater in both groups compared with the relative uninjured nerves.

Stereological analysis confirmed the morphological observations. After the regenerative process, the cross sectional area of both BALB/c and BALB-neuT median nerve was significantly bigger (p<0.05) than the uninjured groups (Fig. 7C). The total number of myelinated fibers was significantly higher (p<0.001) after crush lesion only in the BALB-neuT group. (Fig. 7D). On the other hand, the fiber density decreased in both groups (Fig. 7E), probably due to a larger amount of connective tissue.

As regards axon and fiber size, a significant (p<0.001) difference in comparison to controls was seen only in the BALB-neuT group: mean axon diameter was 21% smaller in regenerated compared to uninjured nerves (2.20±0.10 compared to 2.80±0.22), whereas mean fiber diameter was 26% smaller (2.90±0.15 compared to 3.93±0.35). In addition, the mean myelin thickness significantly decreased in both BALB/c and BALB-neuT groups in comparison to controls: mean myelin thickness of the regenerated fibers in BALB/c mice was 0.44±0.04, in comparison with 0.61±0.03 of uninjured nerves, whereas in BALB-neuT group the difference between regenerated and uninjured myelin thickness was 0.35±0.04 vs 0.56±0.08 (Fig. 7F). The same was true for mean *g-*ratio that showed a significant (p<0.05) difference for both regenerated BALB/c (0.74±0.04) and BALB-neuT (0.74±0.02) mice compared to relative uninjured nerves (respectively 0.69±0.01 and 0.71±0.02) (Fig. 7G–H).

**Figure 8:**
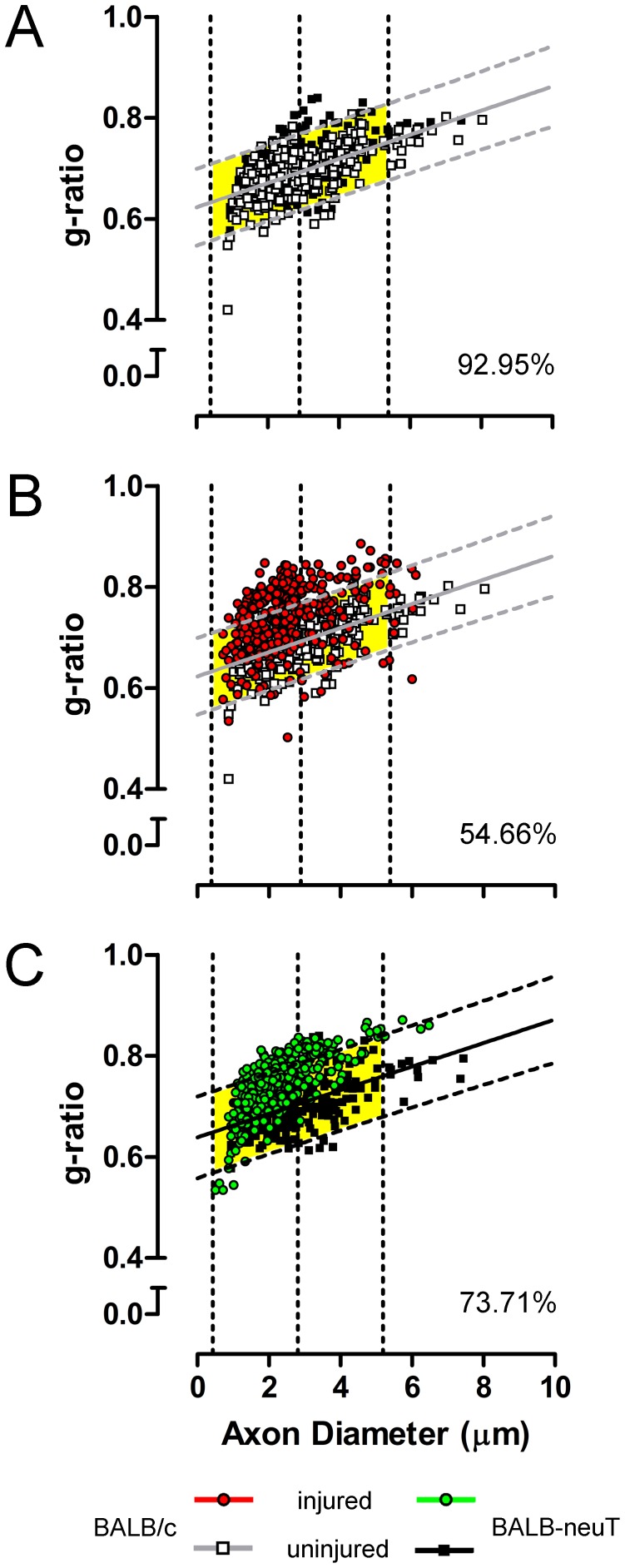
BALB-neuT regenerated fibers have a better fitting than those of BALB/c in their uninjured predictive area. Comparison of BALB-neuT with BALB/c uninjured fibers (A) and respective injured versus uninjured condition (B, BALB/c; C, BALB-neuT). Yellow fields correspond to the predictive area of reference condition (A-B, BALB/c uninjured; C, BALB-neuT uninjured) bounded by 95% prediction interval of regression line and ±2σ (2 standard deviations –95% of cases) of axon diameter. Percentages represent the fiber amount of considered group fitting the predictive area.

In order to explain the enhanced function recovery seen in BALB-neuT group, we have evaluated the percentage of myelinated fibers falling inside a *g*-ratio/axon diameter predictive area in which fibers are considered as functional. By the superposition of uninjured BALB-neuT and uninjured BALB/c scatterplots, we have found that the 92.95% of BALB-neuT fibers reside into the BALB/c predictive area (Fig. 8A) that is consistent with the absence of morphological and functional differences between the two groups.

On the other hand, injured BALB/c fibers fit only for the 54.66% (Fig. 8B), while injured BALB-neuT for the 73.71% (Fig. 8C) within respective uninjured predictive areas. Despite the number of fibers outside the areas are highly significant (both P<0.001), injured BALB-neuT fits significantly better than injured BALB/c group (P<0.001), that is consistent with the enhanced function recovery seen for this group by means of grasping test.

**Figure 9:**
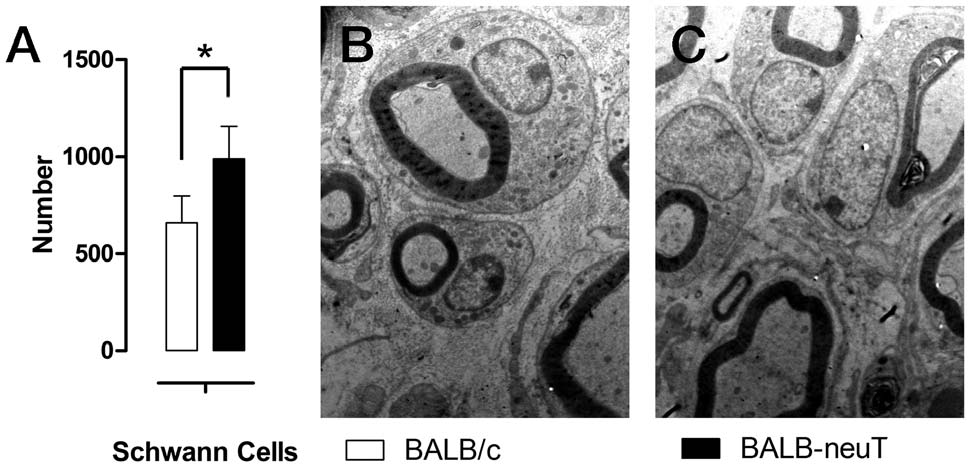
The number of Schwann cells is higher in BALB-neuT mice after regeneration. A, histognams showing the number of Schwann cells counted at electron microscopy. B, C representative electron images of transverse sections of regenerated median nerves of BALB/c and BALB-neuT mice respectively. Magnification: 10000×. Statistical significance: *p<0.05.

Finally, we evaluated the number of Schwann cell nuclei by electron microscopy analysis. Results showed that the number of Schwann cells was significantly higher in transgenic animals compared to wild type (Fig. 9).

### In Response to Peripheral Nerve Injury, the Increase in mRNA for Soluble-NRG1 is Higher in BALB-neuT Mice

To determine whether the over-expression of ErbB2 has effects on the NRG1/ErbB system, we compared the pattern of expression of ErbB receptors (ErbB1, ErbB2, ErbB3 and ErbB4) and of different isoforms of NRG1 (NRG1-alpha, NRG1-beta, soluble NRG1-I/II and transmembrane NRG1-III) by quantitative real time PCR during the early phases following the nerve injury, in BALB/c and BALB-neuT mice.

**Figure 10:**
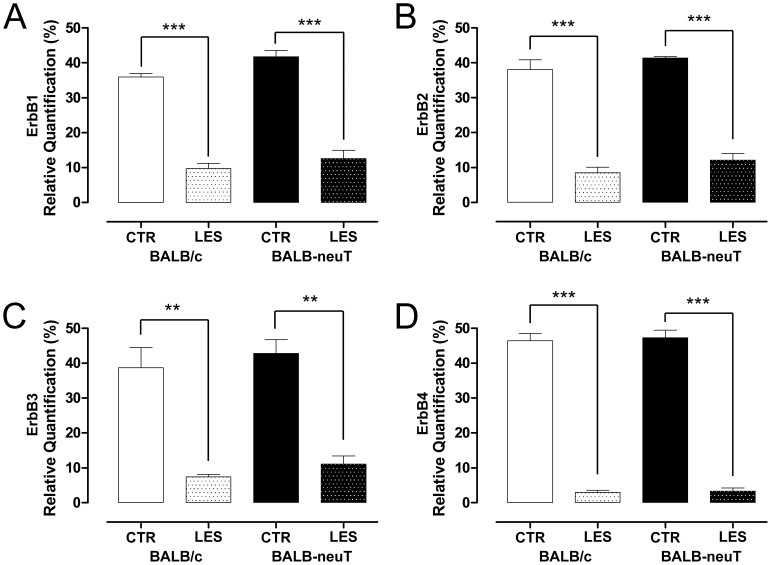
ErbB expression decreases following lesion, and is not influenced by rat ErbB2 over-expression. The relative quantification of different ErbB receptors was obtained by quantitative real-time RT-PCR: data were normalized to the geometric mean of six endogenous housekeeping genes (TBP, UbC, 18S, GAPDH, HPRT1 and MAPK6) and expressed as percentage. Values in the graphics are expressed as mean+SEM. Statistical analysis demonstrates that ErbB1, ErbB2, ErbB3 and ErbB4 expression level significantly decreased two days after the crush lesion (**p<0.01, ***p<0.001). No significant differences were observed between BALB/c and BALB-neuT mice both before and after the injury.

**Figure 11:**
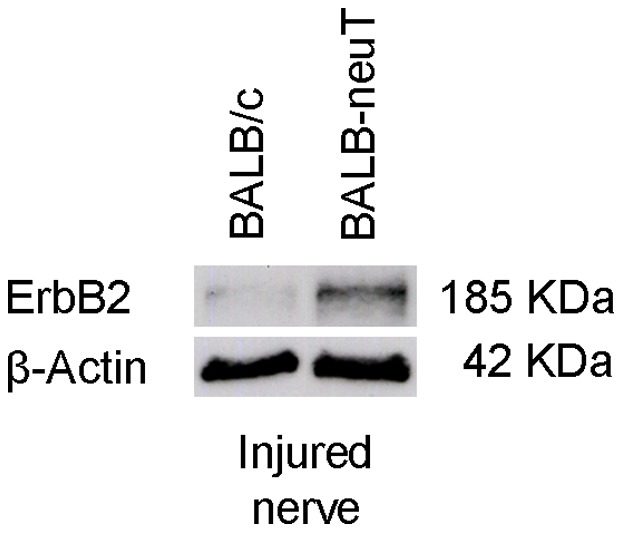
2 days after the crush injury, BALB-neuT mice express higher levels of ErbB2 protein. Western blot analysis showing ErbB2 expression in crushed nerves 2-days after injury. β-actin was used as a loading control. The ErbB2 expression is higher in BALB-neuT mice compared to BALB/c mice.

For BALB-neuT mice, two different primer pairs were designed and validated for their specificity to amplify only the exogenous rat ErbB2, or only the endogenous murine ErbB2. As expected, we detected a high level of rat-ErbB2 in the median, ulnar and radial nerves of BALB-neuT but not in BALB/c mice (data not shown). The expression of all the ErbB receptors was similar between uninjured BALB/c and BALB-neuT mice. Two days after the crush lesion, the mRNA level of the ErbB receptors significantly decreased, both in BALB/c and BALB-neuT mice (Fig. 10A–D). Since the degree of reduction in mRNA levels was similar, we suggest that exogenous ErbB2 over-expression – that we appreciated also in injured animals (Fig. 11) – does not influence the regulation of endogenous ErbB receptor expression before and after the injury.

Statistical analysis performed with two-way ANOVA of all ErbBs showed highly significant main effects of treatment, while the over-expression of rat ErbB2 and the interaction between treatment and ErbB2 over-expression was not significant (Table 2).

**Table 2:** Two-way ANOVA statistical analysis.

factor	TREATMENT			ErbB2 over-expression			TREATMENT vs		
	(CTR vs LES)			(WT vs ErbB2)			ErbB2 over-expression		
	F	df, E	P	F	df, E	P	F	df, E	P
**ErbB1**	249.377	1, 8	0.000	6.078	1, 8	0.059	0.725	1, 8	0.419
**ErbB2**	242.377	1, 8	0.000	3.348	1, 8	0.105	0.009	1, 8	0.927
**ErbB3**	73.720	1, 8	0.000	1.115	1, 8	0.322	0.145	1, 8	0.954
**ErbB4**	756.713	1, 8	0.000	0.158	1, 8	0.702	0.026	1, 8	0.876
**NRG1-I/II**	101.274	1, 8	0.000	12.432	1, 8	0.008	13.925	1, 8	0.006
**NRG1-III**	28.303	1, 8	0.001	3.903	1, 8	0.084	1.351	1, 8	0.279
**NRG1-alpha**	261.214	1, 8	0.000	29.631	1, 8	0.001	22.450	1, 8	0.001
**NRG1-beta**	133.593	1, 8	0.000	24.080	1, 8	0.001	19.388	1, 8	0.002

For quantitative real time PCR data, the effect of treatment (CTR versus LES), the effect of ErbB2 over-expression (BALB/c versus BALB-neuT) and the interaction between the two factors (treatment and ErbB2 over-expression) were analyzed by two-way ANOVA test. Analysis of all ErbBs showed highly significant main effects of treatment (CTR versus LES) while the effect of ErbB2 over-expression (BALB/c versus BALB-neuT) and the interaction between the two factors (treatment and ErbB2 over-expression) was not significant. Analysis of NRG1 showed highly significant main effects of treatment (CTR versus LES) for all NRG1 isoforms analyzed; the effect of ErbB2 over-expression (BALB/c versus BALB-neuT) and the interaction between the two factors (treatment and ErbB2 over-expression) were highly significant only for NRG1 alpha, beta, type I/II. (df = degrees of freedom, E = error, P = P-value).

**Figure 12:**
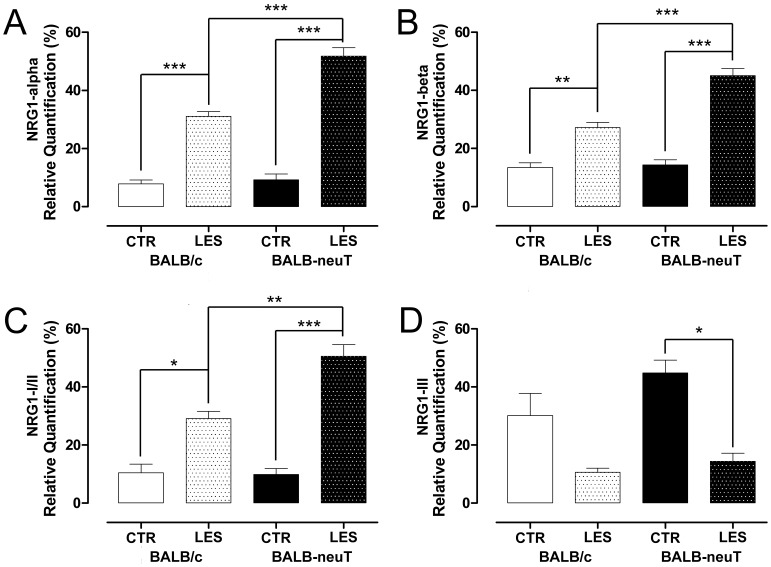
Soluble NRG1 expression increases following lesion, and its expression is positively influenced by ErbB2 over-expression. The relative quantification of different NRG1 isoforms was obtained by quantitative real-time RT-PCR. Results were normalized to the geometric mean of six endogenous housekeeping genes (TBP, UbC, 18S, GAPDH, HPRT1 and MAPK6) and expressed as percentage. Values in the graphics are expressed as mean+SEM. Statistical analysis demonstrates that NRG1-alpha, NRG1-beta and NRG1-I/II mRNA expression significantly increased two days after the crush lesion (*p<0.05, **p<0.01, ***p<0.001) both in BALB/c and BALB-neuT animals. Interestingly, in the BALB-neuT mice there was a significantly higher increase of mRNA expression compared to BALB/c animals. NRG1-III mRNA decreases after the crush injury, but only in the BALB-neuT mice.

On the other hand, NRG1-alpha, NRG1-beta and NRG1-I/II mRNA expression was similar in mice not undergoing the crush lesion for both groups (Fig. 12A–C). After the injury, the expression of these NRG1 isoforms increased in both BALB/c and BALB-neuT mice. Interestingly, after injury, BALB-neuT mice showed a significantly higher level of NRG1-alpha, NRG1-beta and NRG1-I/II mRNA expression in comparison to BALB/c mice after injury. On the contrary, NRG1-III mRNA expression significantly decreased after the injury only in the BALB-neuT mice (Fig. 12D).

Statistical analysis performed with two-way ANOVA showed highly significant main effects of treatment for all NRG1 isoforms analyzed; the effect of the ErbB2 over-expression and the interaction between treatment and over-expression of rat ErbB2 were highly significant only for NRG1 alpha, beta, type I/II (Table 2).

## Discussion

The availability of transgenic and knockout mouse models has opened exciting perspective in the study of peripheral nerve repair and regeneration [3], [4]. ErbB/NRG signaling is involved in several important aspects in the development and regeneration of peripheral nerves [45]. ErbB2 null mutant mice die at midgestation because of heart malformation [46] but important alterations of nervous system occur (marked reduction of Schwann cell precursors, which accompany the spinal nerves at the embryo stage E10.5, and severe hypoplasia of cranial sensory and sympathetic ganglia) [47]. Experiments with conditional ablation of ErbB2 in late Schwann cell development show a lack of Schwann cells and poorly fasciculated and disorganized nerves [47]. Moreover, Morris and co-workers [48] demonstrated that Schwann cell precursors are present and are proliferative in the dorsal root ganglia of transgenic mice lacking ErbB2/ErbB3, but they fail to migrate to the periphery.

Using inducible Krox20-Cre to ablate ErbB2 in immature Schwann cells, it has been shown that the thickness of the myelin sheath is reduced and contains fewer myelin wraps compared with wild-type mice [9]. When the ablation of ErbB2 gene occurs in adult Schwann cells, no apparent effect on the maintenance of already established myelinated peripheral nerves is seen after ErbB2 gene reduction [45]. Moreover, after a peripheral nerve injury in Krox20-Cre ErbB2 mice, there is an increased Schwann cell proliferation and cell death compared to controls. In contrast, ablating ErbB2 exclusively in adult Schwann cells has no detectable effect on survival and cell division after injury. Thus, Schwann cell responses to axotomy depend on the timing of ErbB2 ablation [45].

While the effects of ErbB2 deletion in the peripheral nervous system have already been analyzed, the consequences of its over-expression have not been investigated to date. To fill this gap, we examined the effect of the over-expression of ErbB2 on healthy nerve and its role on nerve regeneration after a crush injury of the median nerve.

We examined the peripheral nerves of healthy adult mice and we did not detect morphological and stereological differences between mice of the same offspring over-expressing (BALB-neuT mice) or not (BALB/c mice) the neu/ErbB2 gene; indeed, nerve cross sectional area, number of myelinated fibers and fiber density, as well as myelinated fibers size, myelin thickness and *g-*ratio were comparable. These data suggest that ErbB2 over-expression does not influence murine peripheral nerve phenotype. By contrast, the over-expression of ErbB2 appeared to speed up nerve regeneration after damage, as shown by a faster functional motor recovery assessed with the grasping test. Yet, stereological analysis showed that regenerated myelinated fibers analyzed at light microscopy on semithin sections are, on average, more numerous, smaller and with thinner myelin sheath in BALB-neuT mice, suggesting that ErbB2 over-expression induces a richer posttraumatic axonal sprouting. We also tried to count unmyelinated fibers at electron microscopy. However, in regenerated nerves it was not possible to recognize the single small axons within the Remak bundles. Thus, we cannot exclude the hypothesis that the observed higher number of myelinated axons is due to a shift between unmyelinated to myelinated axons.

Since it has been shown that NRG1 stimulates the growth of Schwann cells through the activation of ErbB2/ErbB3 heterodimers [49], [50], we hypothesize that the constitutive over-expression of ErbB2 receptor makes Schwann cells more sensitive to NRG1, which is significantly up-regulated already in wild type mice. Up-regulated soluble NRG1 activates ErbB2/ErbB3 and, through a positive feedback loop (helped by ErbB2 overexpression), augments its expression stimulating Schwann cell proliferation [51], [52], [53], [54]. Higher Schwann cell proliferation, confirmed by electron microscopy quantification, could be at the basis of the changes that we observed during posttraumatic nerve regeneration. Nevertheless, we can’t exclude the possibility that some of the effects observed in the transgenic mice are due to enhanced ErbB2 signaling also in the axons.

To analyze the expression level of all ErbB family members and of the different NRG1 isoforms, we performed relative quantification using real time qPCR analysis. Peripheral nerve injury interferes with the expression of many genes: the identification of the optimal reference gene (whose expression level is constant in both control and injured samples) is therefore an important issue to deal with. Taking inspiration from the literature about accurate normalization [55], [56] we identified six good candidate housekeeping genes whose expression is expected to be stable even after nerve injury and we normalized the gene expression of ErbB and NRG1 genes to the geometric average of these six housekeeping genes.

We observed a strong significant decrease of mRNA expression of all ErbB receptors in lesioned mice, both in BALB/c and BALB-neuT mice.

The down-regulation of ErbB receptors is in contrast with data of other authors [57]. However, this discrepancy can be explained since our injury model and our analysis conditions are different: they performed a surgical transection, while we carried out a crush lesion; they observe a protein increase 5 days after axotomy, while we are analyzing transcript expression 2 days after the crush injury. The stability of protein and transcript can be different and a decrease in mRNA at 2 days is not incompatible with an increase of protein at 5 days.

We observed also down-regulation of transmembrane-type III NRG1 in transgenic mice. Anyway, the expression level of this isoform in our samples was already really low, as suggested by the high threshold cycle of this isoform achieved in the qPCR reaction; indeed, the transmembrane isoform is axonal and when we extract RNA from the nerve, the majority derives from Schwann cells. In its place, a strong significant up-regulation of mRNA coding for soluble-type I/II NRG1 and for the alpha and beta isoforms (that can be associated with both soluble and transmembrane NRG1 isoforms) can be observed in lesioned animals. Intriguingly, we found that in BALB-neuT mice the soluble NRG1 up-regulation, both alpha and beta, is significantly higher than in BALB/c mice. We verified that BALB-neuT mice injured nerves overexpress ErbB2 protein and we hypothesize that soluble NRG1 (whose amount increases following nerve lesion), by binding overexpressed ErbB2/ErbB3, activates a positive feed-back loop, that could lead to the higher level of NRG1 expression observed in BALB-neuT mice [51], [52], [53], [54]. Anyway, we cannot exclude the possibility that upregulation of the soluble isoform of NRG1 is responsible also of the thinner myelin sheath observed in BALB-neuT versus BALB/c mice after injury (27).

Further studies are required to explain in more detail the role played by NRG1 in nerve regeneration in over-expressing ErbB2 mice. However, our data support the view that the stimulation of Schwann cells by soluble NRG1 is responsible for the faster recovery observed in transgenic mice following nerve injury. This might open interesting perspectives for innovative pharmacological strategies for improving the clinical outcome of peripheral nerve lesions.
